# TRIB1 is a positive regulator of hepatocyte nuclear factor 4-alpha

**DOI:** 10.1038/s41598-017-05768-1

**Published:** 2017-07-17

**Authors:** Sébastien Soubeyrand, Amy Martinuk, Ruth McPherson

**Affiliations:** Atherogenomics Laboratory, University of Ottawa Heart Institute, Ottawa, Canada

## Abstract

The *TRIB1* locus has been linked to both cardiovascular disease and hepatic steatosis. Recent efforts have revealed TRIB1 to be a major regulator of liver function, largely, but not exclusively, via CEBPA degradation. We recently uncovered a functional interaction between *TRIB1* and *HNF4A*, another key regulator of hepatic function, whose molecular underpinnings remained to be clarified. Here we have extended these findings. In hepatoma models, HNF4A levels were found to depend on TRIB1, independently of its impact on *CEBPA*. Using a reporter assay model, MTTP reporter activity, which depends on HNF4A, positively correlated with TRIB1 levels. Confocal microscopy demonstrated partial colocalization of TRIB1 and HNF4A. Using overexpressed proteins we demonstrate that TRIB1 and HNF4A can form complexes *in vivo*. Mapping of the interaction interfaces identified two distinct regions within TRIB1 which associated with the N-terminal region of HNF4A. Lastly, the TRIB1-HNF4A interaction resisted competition with a CEPBA-derived peptide, suggesting different binding modalities. Together these findings establish that *TRIB1* is required for *HNF4A* function. This regulatory axis represents a novel *CEBPA*-independent aspect of *TRIB1* function predicted to play an important role in liver physiology.

## Introduction

The TRIBBLES proteins form a family of 3 mammalian proteins (TRIB1, 2 and 3) sharing ~30% overall identity, characterized by the presence of a relatively well-conserved core kinase-like domain and divergent N and C-termini. Unlike *bona fide* kinases, the TRIBBLES exhibit no detectable kinase activity (with the exception of TRIB2) as a result of mutations at key catalytic residues^1, 2^. Rather, they act as molecular adaptors by regulating other kinases and/or targets thereof. Over the years numerous roles, pathological and physiological roles have been ascribed to the TRIBBLES^3, 4^. For *TRIB1* specifically, research has focused on leukemia and more recently, lipid and lipoprotein metabolism. Genome-wide Association Studies (GWAS) have identified a locus proximal (~30 kb) to *TRIB1* that associates with increased plasma triglycerides and a predisposition for cardiovascular disease (CAD)^5^. Importantly, TRIB1 has also been linked to hepatic steatosis^6^. In a previous work we observed an inverse correlation between the top CAD risk single nucleotide polymorphism (SNP) and *TRIB1* expression levels in whole blood on the one hand and circulating lipids on the other hand, suggesting that TRIB1 may play a role in reducing hepatic triglyceride synthesis and secretion in humans^7^.

Whole animal models have uncovered roles for TRIB1 in both lipid and glucose metabolism^8^. Of the numerous proximal targets of TRIB1 identified over the years, there is a consensus on the ability of TRIB1 to promote CEBPA degradation^9^. Recently Bauer *et al*. have demonstrated a close functional relationship between these proteins in mouse liver^10^. More specifically, liver-specific *TRIB1* deficiency could be partially rescued by *CEBPA* knock-out hinting that a major function of TRIB1 in the liver is to regulate CEBPA. Importantly, while circulating lipid levels could be rescued by *CEBPA* knock-out, hepatic lipid accumulation (steatosis) could not, indicating that *TRIB1* has roles transcending *CEBPA* regulation.

We recently identified a functional interaction between *HNF4A* and *TRIB1* ^11^. *TRIB1* suppression resulted in impaired *HNF4A* function inferred from reduced *HNF4A*, *HNF1B* and increased *SNAI1* transcripts in primary hepatocytes. In HepG2 cells, a widely used hepatic cell model, HNF4A protein levels were reduced as a result of *TRIB1* suppression while *HNF4A* suppression increased *TRIB1* transcript abundance. HNF4A is a highly conserved member (NR2A1) of the nuclear receptor family and is unique among the nuclear receptor superfamily in its ability to bind DNA exclusively as a homodimer and activate transcription in the absence of exogenous ligand^12^. HNF4A plays a pivotal metabolic role by regulating the expression of liver and intestinal genes^13, 14^. HNF4A is essential for TG, cholesterol homeostasis and bile acid metabolism and helps regulate the expression of several key lipoprotein regulators including *APOC3* and *MTTP* ^15–19^. In addition, loss of HNF4A perturbs the function of key regulators of the mesenchymal-to-epithelial transition (EMT) and is associated with the development of hepatic steatosis and hepatocellular carcinoma^20, 21^. Interestingly HNF4A and CEBPA co-localize extensively on chromatin and loss of *Hnf4a* reduces the ability of Cebpa to bind DNA and vice versa^22^. In this work the interplay between *TRIB1* and *HNF4A* is explored and a general requirement for the *TRIBBLES* in sustaining HNF4A protein levels is demonstrated. In addition a protein-protein interaction between HNF4A and TRIB1 is described and mapped.

## Results

### *TRIB1* regulates *HNF4A* in HuH-7 hepatoma cells

**Figure 1:**
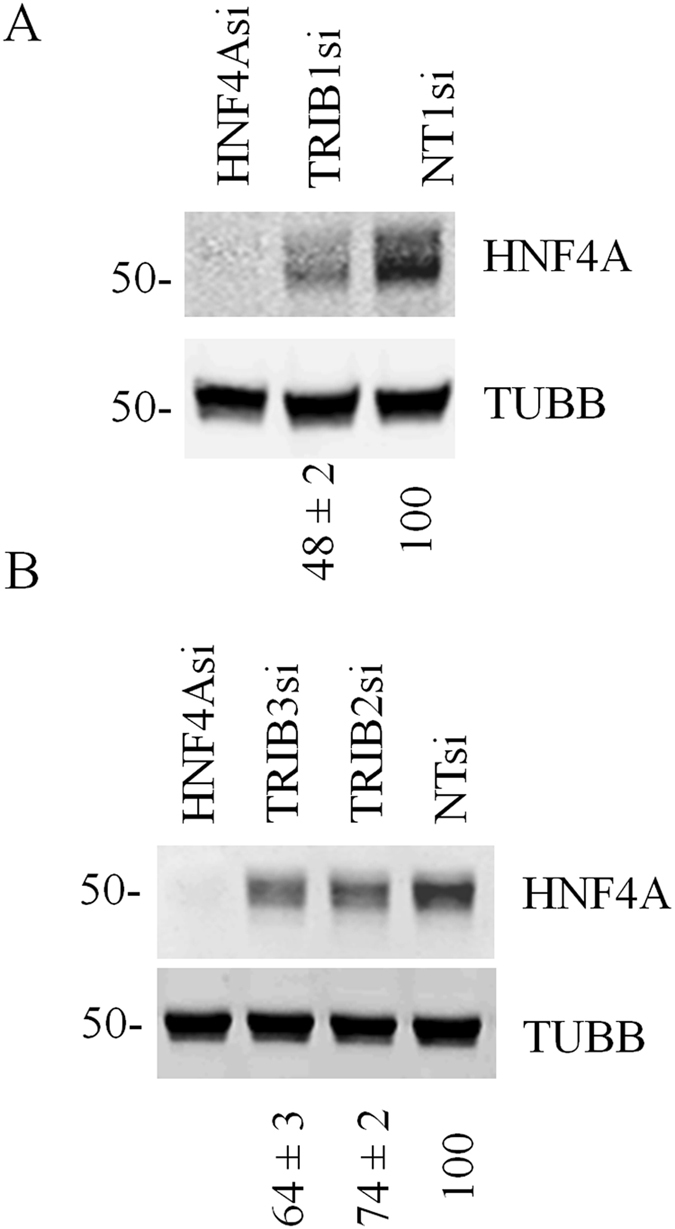
HNF4A expression depends on all the *TRIBBLES*, with a greater contribution of TRIB1. (**A,B**) HuH-7 cells were treated for 72 h with the indicated siRNAs and analyzed for protein content by Western blotting. Quantifications of HNF4A (relative to TUBB and then normalized to the NT value) are shown under the blot (means of 3 biological replicates ± S.D). Changes in HNF4A transcript were tested for statistical significance using one-way ANOVA followed by between group comparisons using Tukey’s post-hoc test. TRIB1 vs TRIB2/TRIB3: p < 0.01; TRIB1/2/3 vs NT: p < 0.001.

In our previous work we observed that *TRIB1* suppression led to reduced *HNF4A* expression in both HepG2 cells and human primary hepatocytes^11^. Interestingly while TRIB1 suppression is associated with reduced *HNF4A* transcript levels in primary hepatocytes, no such change is obvious in HepG2 (Suppl Fig. 1), suggesting that TRIB1 may utilize transcriptional and non-transcriptional mechanisms to regulate HNF4A. To examine how prevalent this relationship was, we examined the impact of *TRIB1* silencing in another widely studied human hepatoma cell line, HuH-7 cells where *TRIB1* suppression led to reduced HNF4A protein (Fig. 1A). This change was associated with a 26% reduction in *HNF4A* transcript (74 ± 18% of control (n = 6, p = 0.02). Thus HuH-7 cells, in contrast to HepG2 cells, seem to have retained some capacity to sustain *HNF4A* transcript levels via *TRIB1*. Yet as HNF4A protein levels in HuH-7 and HepG2 cells exhibit similar and pronounced (~50% reduction and^11^) sensitivities to *TRIB1* silencing, this suggests that transcriptional impacts may not single-handedly account for lower HNF4A protein expression in HuH-7 cells.

### Maintaining HNF4A levels requires the three *TRIBBLES*, but most prominently TRIB1

We previously noted that *TRIB1* suppression in primary hepatocytes and HepG2 cells resulted in higher levels of the other *TRIBBLES* (*TRIB2* and *TRIB3)* ^11^, which could indicate that *TRIB2* and *TRIB3* are functionally linked to *HNF4A* as well; similarly, *TRIB2* and *TRIB3* transcripts were increased in HuH-7 cells upon TRIB1 silencing (data not shown). To investigate possible contributions of the other *TRIBBLES* in controlling HNF4A levels. *TRIB2* and *TRIB3* mRNA were targeted by their cognate siRNAs. As seen with *TRIB1*, *TRIB2* and *TRIB3* silencing also reduced HNF4A protein levels (Fig. 1B), albeit more modestly suggesting that all *TRIBBLES* contribute to maintain HNF4A steady state levels, with *TRIB1* playing a prominent role. While validation at the RNA level confirmed that the corresponding *TRIBBLES* transcripts were reduced, endogenous TRIBBLES proteins could not be detected in cellular extracts by Western blot, in line with low RNA expression (Cp values of ~25–30) (Suppl Fig. 2).

TRIB1 and TRIB3 are reportedly unstable proteins and thus reduced mRNA levels are expected to result in reduced protein expression^23^. As instability was assessed using overexpressed proteins as proxies, the fate of the endogenous protein remained unclear however. To address this last point, large scale immunoprecipitations on control and silenced lysates were undertaken; we decided to focus on TRIB1 in view of its greater contribution to HNF4A function. Western blot analysis of a large scale TRIB1 immunoprecipitation confirmed the efficacy of the knock-down at the protein level (Suppl Fig. 2C). This finding, in conjoncution with our earlier findings demonstrating that targeting two distinct regions of the *TRIB1* transcript resulted in reduced HNF4 expression in HepG2 cells, clearly establish that TRIB1 is needed for sustaining HNF4A expression.

### Impact of TRIB1 on HNF4A requires more than CEBPA

**Figure 2:**
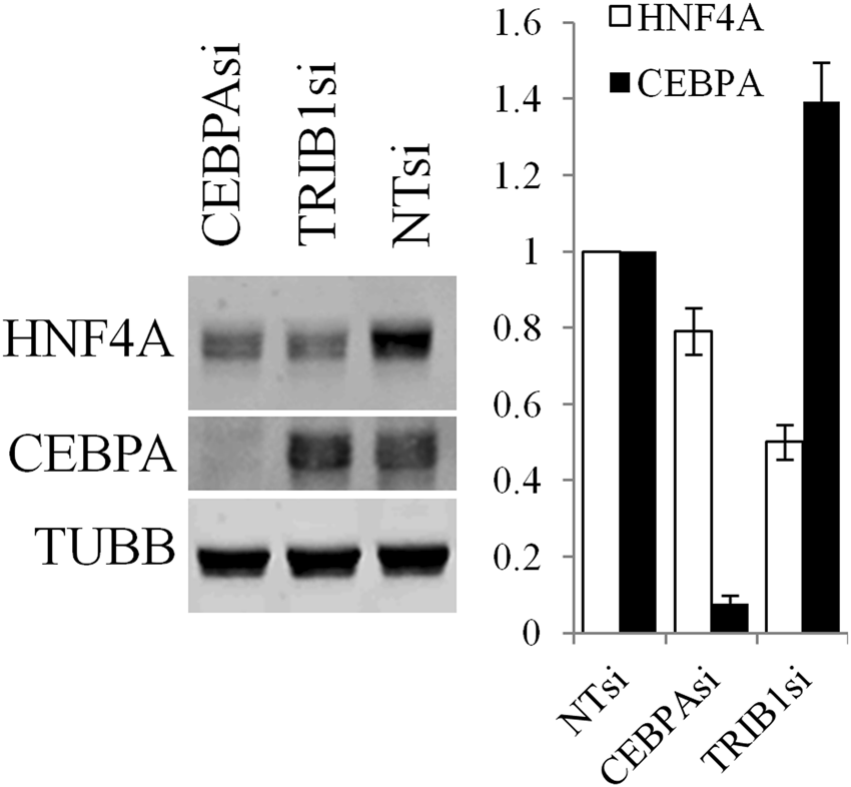
Interplay between TRIB1, CEPBA and HNF4A in HuH-7 cells. HuH-7 cells were silenced with the indicated siRNA for 48 h prior to Western blotting using the indicated antibodies. Representative blot is shown. Quantification of 3 Western blots is shown on the right; bars represent the means of 3 independent biological replicates ± S.D. All changes are statistically significant (p < 0.05) from NTsi.

An important question relates to the role of the CEBPA-TRIB1 axis in HNF4A regulation given that CEBPA and HNF4A are functionally intertwined^22^. While our suppression data suggested that all 3 *TRIBBLES* contribute to the maintenance of HNF4A, recent studies have demonstrated an essential contribution of *Cebpa* in mediating *Trib1* function in murine liver^10^. A major role previously ascribed to TRIB1 and 2, but not shared by TRIB3 is to degrade CEBPA via COP1 recruitment^9^. Thus the contribution of CEBPA to HNF4A expression was tested in HuH-7 cells. *CEBPA* silencing reduced HNF4A levels indicating that *CEBPA* is necessary for maximal *HFN4A* expression (Fig. 2). By contrast, *TRIB1* suppression increased CEBPA protein levels, a finding that is directionally inconsistent with the reduced HNF4A entailed by suppressing *TRIB1*. Thus changes in CEBPA are insufficient to account for the impact of *TRIB1* silencing on HNF4A.

### *TRIB1* silencing affects HNF4A function

**Figure 3:**
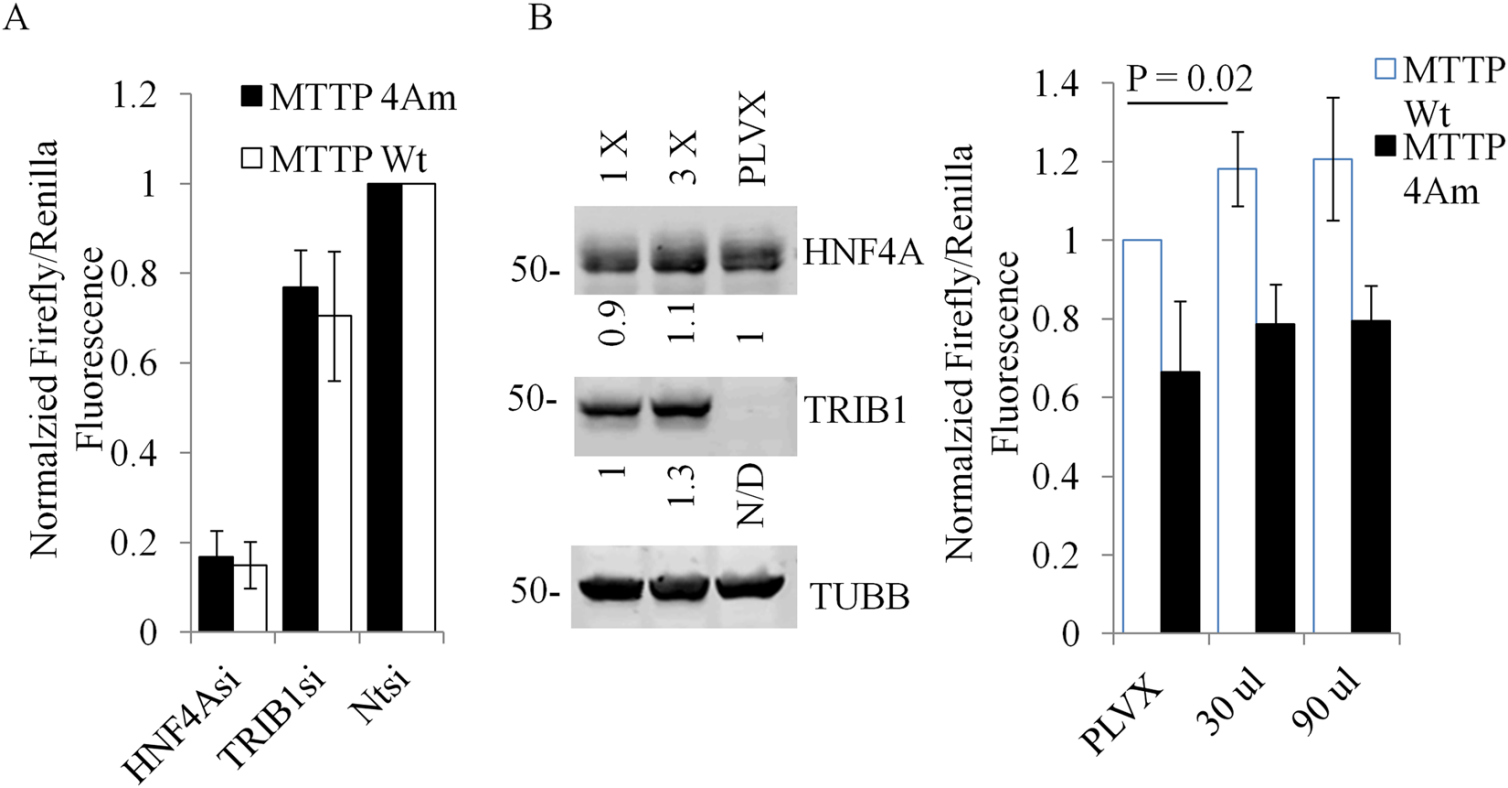
Overexpression of *TRIB1* in HuH-7 cells increases *MTTP* promoter assay activity but does not affect HNF4A protein level. (**A**), HuH-7 cells were treated for 48 h with indicated siRNAs, washed and transfected with MTTP constructs (and Renilla internal control) for an additional 24 h. Samples were lysed in Passive Lysis Buffer and processed for fluorescence. Results represent the means of 3 experiments (±S.D). Values are normalized to the Non-Target (NT) siRNA-treated samples. (**B**), A stable pool of HIS-TRIB1 expressing cells was generated by transduction with increasing viral titer (1X, 3X). A control pool was generated using the empty vector (PLVX at 3X titer). Cells transfected with *MTTP* promoter and renilla internal control constructs for 24 h were lysed and analyzed by Western blotting (left) or luciferase assay (right). Data represent the mean of 5 experiments normalized to PLVX (Wt MTTP) values, ±S.D. Quantifications of the Western blot are relative to TUBB and normalized to either PLVX (HNF4A) or 1X MOI (TRIB1). Statistical significance was assessed using the Student’s 2-tailed t-test.

Next, the impact of *TRIB1* suppression on HNF4A function was examined using a *MTTP* promoter reporter assay previously demonstrated to depend on HNF4A for maximal activation^17^. In HuH-7 cells *HNF4A* silencing reduced *MTTP* promoter (MTTPp) activity to about 10–15% of its maximal value (Fig. 3A), consistent with its strong dependence on HNF4A. Surprisingly a mutated construct that is deficient for HNF4A binding (MTTP4Am), which reduced MTTPp activity by ~35% relative to the Wt construct in naïve cells (data not shown), was similarly sensitive to *HNF4A* suppression (Fig. 3A). Thus, in HuH-7 cells at least, depletion of *HNF4A* seems to affect MTTP promoter activity via two distinct mechanisms: proximally, through loss of direct binding of HNF4A (i.e. about 35% of the total signal), as well as distally, possibly through a reprogramming of the cell transcriptional machinery. Be that as it may *TRIB1* suppression decreased promoter activities of both constructs by ~30%, a change overall consistent with the HNF4A protein reduction observed. Thus, *TRIB1* silencing decreases HNF4 protein level and *HNF4A*-dependent promoter activity.

To assess the impact of *TRIB1* overexpression, stable pools of TRIB1 (tagged with a C terminal His tag) were obtained by transducing HuH-7 cells with lentiviral constructs driving *TRIB1* expression. Unlike its suppression, introduction of exogenous TRIB1 had no noticeable impact on HNF4A levels (Fig. 3B). When MTTPp activity was measured however, *TRIB1* overexpression was associated with a modest but statistically significant increase in Wt promoter activity, in line with increased HNF4A function (Fig. 3B).

### TRIB1 co-localizes partially with HNF4A

**Figure 4:**
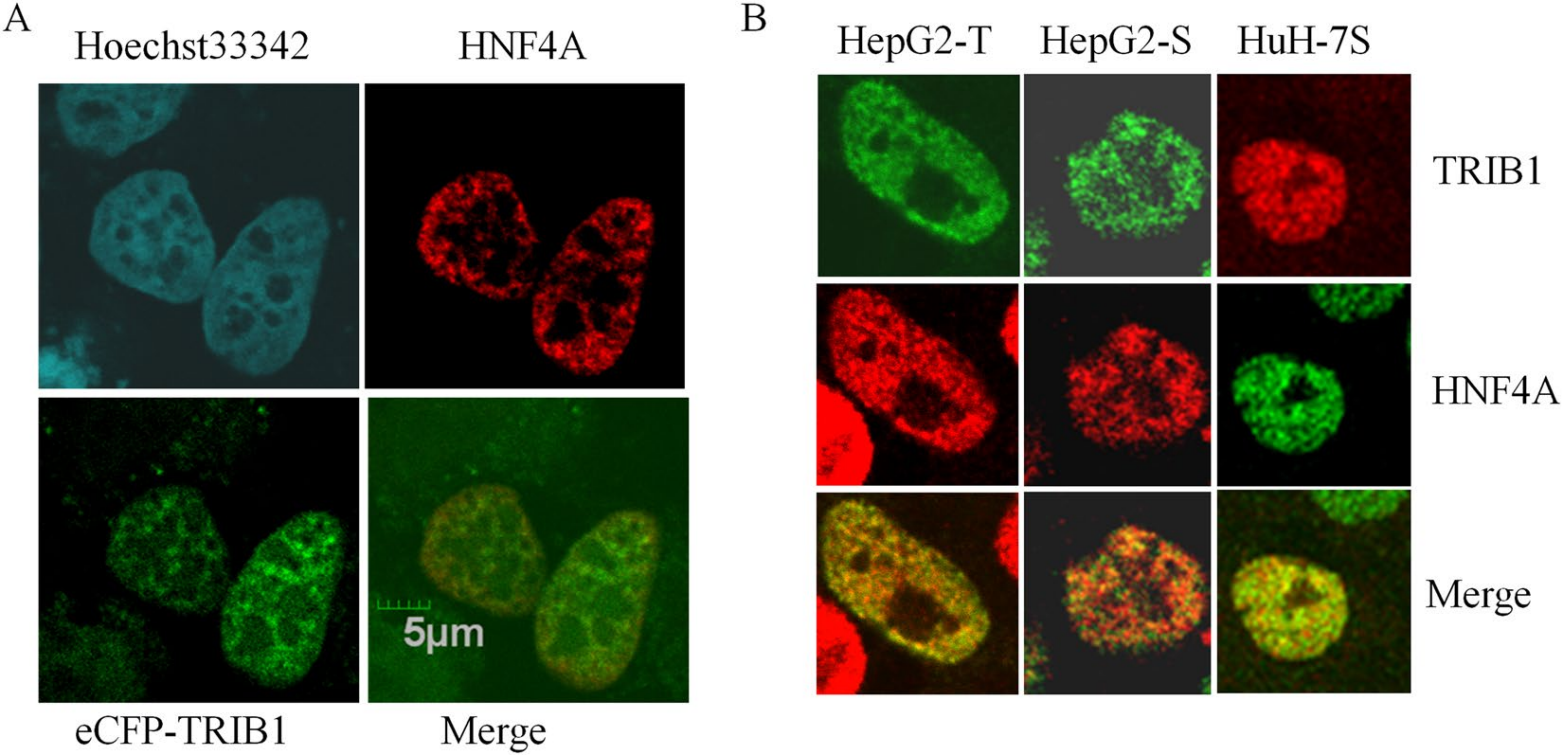
Partial co-localization of TRIB1 and HNF4A *in vivo*. Confocal microscopy of HNF4A- and TRIB1- expressing cells. (**A**), HeLa cells co-transfected with plasmids encoding *HNF4A* and *ECFP-TRIB1* for 48 h were fixed, permeabilized, incubated with HNF4A-specific antibodies and visualized by microscopy. (**B**), Expression in hepatocyte models. Cells were either transfected for 48 h with a plasmid encoding for FLAG-TRIB1 (HepG2-T) or transduced stably with TRIB1 (HepG2-S) or HisTRIB1 (HuH-7S) prior to microscopy. Immunocytochemistry was performed with HNF4A and antibodies specific for either FLAG (HepG2-T) or TRIB1 (HepG2-S, HuH-7S).

The experiments above indicated that *TRIB1*, may regulate *HNF4A* via multiple pathways. One possible route may involve a direct effect of TRIB1 on HNF4A. Indeed, TRIB1 was previously shown to interact with RAR^24^, a distantly related nuclear receptor. The localization of both proteins was examined by confocal microscopy, with the expectation that co-localization events represent putative sites of functional convergence and association. Microscopy was performed in several cell types, using either endogenous HNF4A or exogenous HNF4A, in the presence of overexpressed TRIB1; in all cell types examined, endogenous TRIB1 protein was undetectable, defined by a resistance of detectable signal to several *TRIB1* siRNA and/or antisense oligonucleotide treatments (data not shown). Both proteins showed qualitatively comparable staining: pan-nuclear signals with some granularity (Fig. 4 and Suppl Figs 3 and 4). Although the signals were largely distinct spatially, consistent with independent functions, some co-localization visualized by orange signals were also observed, indicating that a subset of these proteins have the potential to converge functionally *in situ*.

### TRIB1 forms a complex with HNF4 *in vivo*

**Figure 5:**
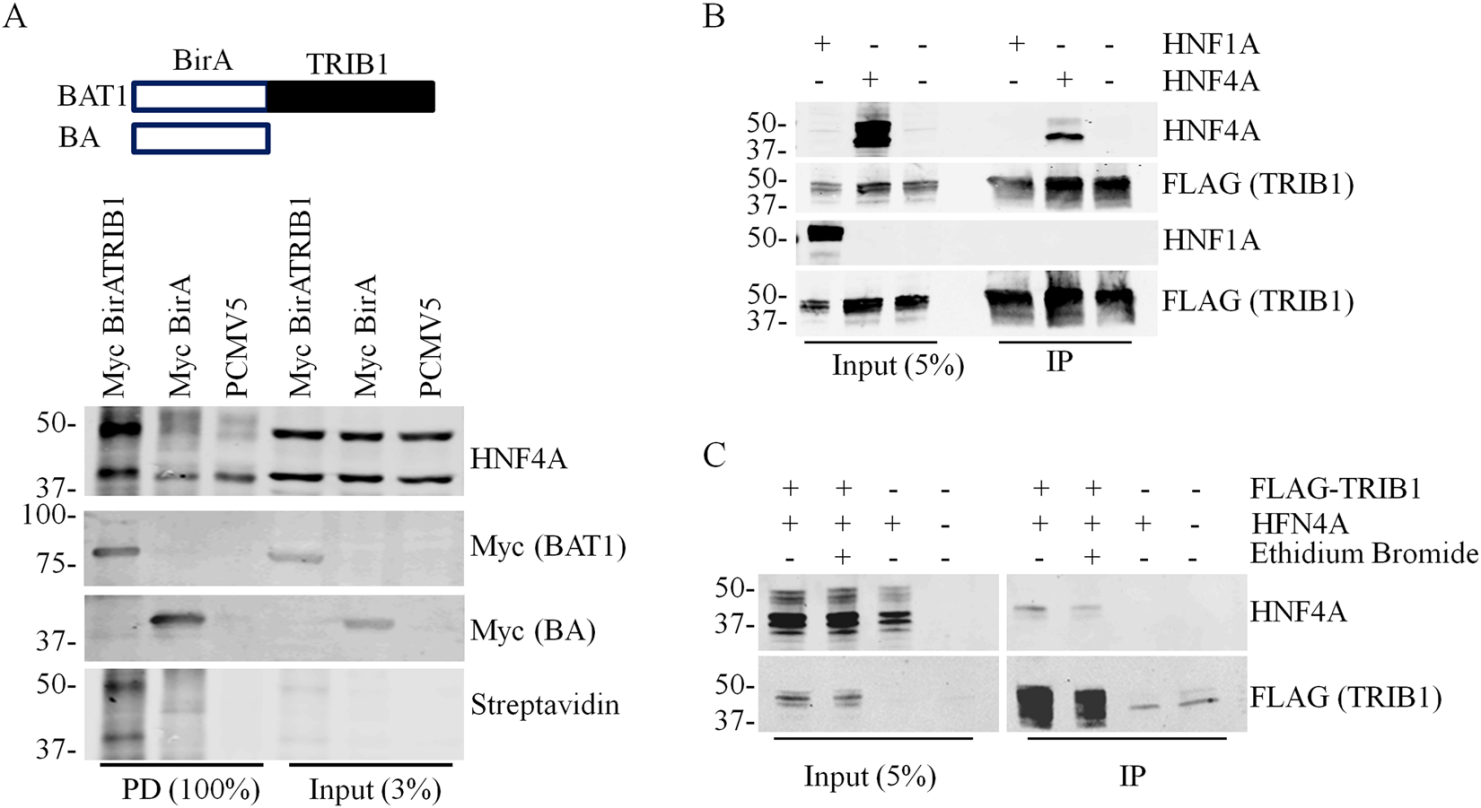
TRIB1 and HNF4A form a complex. (**A**), Constructs coding for myc-tagged BirA alone or inserted N terminal of TRIB1 were co-transfected with HNF4A expression plasmids in HEK293T for 24 h. Following an additional 24 h growth in media supplemented with 0.1 mM biotin, cells were harvested and lysed under denaturing conditions. Biotinylated targets were then isolated using streptavidin beads and analyzed by Western blotting using cognate antibodies followed by streptavidin coupled to IRDye800CW. (**B**), Plasmids encoding either *HNF4A* or *HNF1A* were transfected alongside FLAG-tagged *TRIB1* plasmids in HEK293T cells. Potential complexes were isolated with FLAG-specific immunobeads and analyzed by Western blotting. (**C**), Interaction is independent of DNA binding. Ethidium bromide (0.2 mg/ml) was included during the immunoprecipitation. Samples were analyzed by Western blotting using the indicated antibodies.

While the microscopy indicated that HNF4A and TRIB1 share some common nuclear territories, confocal resolution is insufficient to examine physical interaction. To assess whether HNF4A/TRIB1 can directly interact *in vivo*, we employed the BirA biotin ligase system of Roux *et al*.^25^ which relies on the ability of a fused BirA to tag vicinal proteins with biotin: presence of biotin in a target reflects its propensity to interact with a BirA fusion bait. *TRIB1* tagged at its N-terminus with the BirA moiety (or BirA alone) was co-transfected with a *HNF4A* expression vector in HEK293T cells, which do not express endogenous *HNF4A*. Transfection of *HNF4A* in 293 T cells resulted in the appearance of two major bands at ~48 and 42 kDa, as assessed by Western blot (Fig. 5A). Cells were lysed under denaturing conditions to prevent *ex vivo* biotinylation and the lysates were fractionated using streptavidin beads under stringent conditions. Western blot analyses revealed that co-transfection with BirA-*TRIB1* resulted in the presence of biotinylated HNF4A in the isolate. By comparison, transfection of either control BirA or an empty plasmid resulted in weaker reactive bands. Significantly, unlike seen with BirA-TRIB1 derived lysates, the streptavidin-bound material from either control did not exhibit evident biotinylation of HNF4A as indicated by the absence of matching streptavidin signal. Thus, presence of the TRIB1 moiety permits HNF4A biotinylation, presumably reflecting the formation of TRIB1-HNF4A complexes *in situ*.

### Interaction of TRIB1 with HNF4A is specific and DNA-independent

To obtain additional evidence supporting the formation of complexes in mammalian cells and to gauge the relative strength of the interaction, CoIP experiments were then performed on HEK293T cellular lysates. HEK293T cells were transfected with *HNF4A* and/or *TRIB1* as indicated. HEK293T transiently expressing FLAG-tagged TRIB1 and either HNF4A or HNF1A, a structurally unrelated transcription factor, were subjected to FLAG immunoprecipitation. HNF4A, but not HNF1A, was isolated by the procedure, indicating that TRIB1 and HNF4A can form a specific complex in cellular extracts (Fig. 5B). Moreover the isolation resisted ethidium bromide, an indication that dsDNA binding is not required for complex formation (Fig. 5C).

### Interaction of with HNF4A involves multiple TRIB1 interfaces

**Figure 6:**
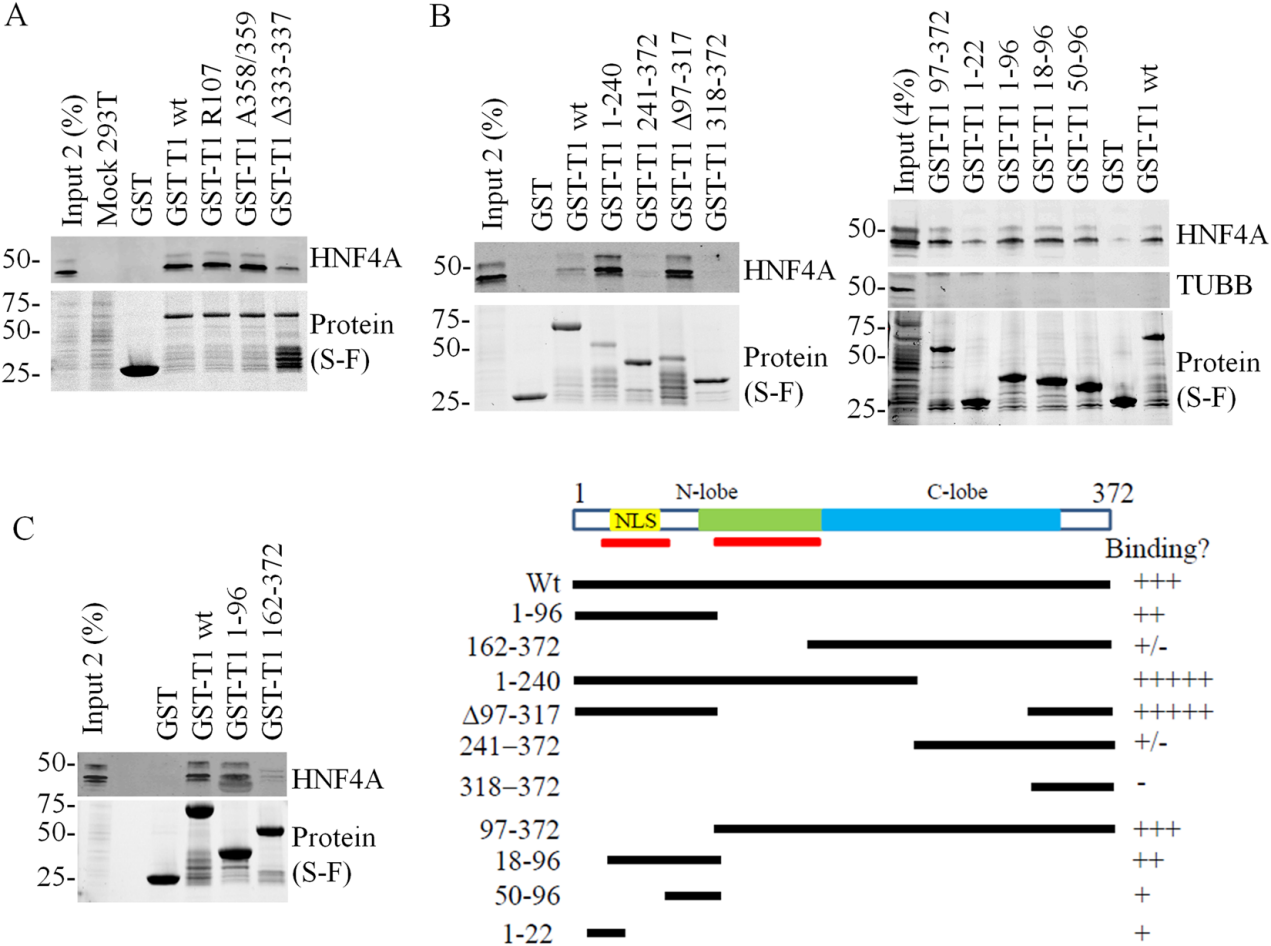
Mapping of the TRIB1 interface associating with HNF4A. Recombinant TRIB1 or fragments thereof, harboring the indicated mutations or deletions were incubated in the presence of HNF4A-expressing HEK293T lysates. **A**, Interaction between TRIB1 and HNF4A does not require MEK1 or COP1 binding interface, nor is it altered by a gain-of-function mutation. **B** and **C**, Multiple TRIB1 epitopes interact with HNF4A *in vitro*. Bound material was isolated and analyzed by Western blot. Corresponding protein baits were imaged prior to transfer using Stain Free gels. Schema illustrates truncations employed and their relative binding to HNF4A.

Next, we explored TRIB1/HNF4A interaction modalities using recombinant TRIB1 protein. GST pull-downs assays performed in HuH-7 and HepG2 cells confirmed the suitability of the method: recombinant TRIB1 could interact with endogenous HNF4A from both cell types (Suppl. Fig. 5). Next, the interaction interface on TRIB1 was investigated using pull-down assays on HEK293T transfected with *HNF4A*; this approach yielded higher HNF4A levels than could be obtained from either HepG2 or HuH-7 cells thereby rendering the analyses more robust. Contributions of the better characterized interaction epitopes on TRIB1, i.e. the COP1 and the MEK1 binding sites were first evaluated. Mutating either site did not prevent HNF4A association indicating that these regions are not required for binding to HNF4A although deleting the MEK1 interaction interface impaired binding (Fig. 6A). Furthermore, a gain of function mutation (L107R)^26^ had no detectable impact on the interaction.

The epitope on TRIB1 responsible for its interaction with RAR, a nuclear receptor distantly related to HNF4A, has been mapped to a large central region of TRIB1 comprising the pseudokinase domain^24^. As this domain is highly conserved among the TRIBBLES, this suggested that other TRIBBLES may also interact with HNF4A. This was tested using TRIB1, TRIB2 and TRIB3 (Suppl Fig. 6). Interestingly all 3 TRIBBLES were found to interact with HNF4A although TRIB1 and TRIB3 displayed higher binding after correcting for the amount of bait. Unexpectedly, unlike observed previously with RAR, the presence of an intact pseudokinase domain is not essential as a *TRIB1* construct missing the core pseudokinase region (Δ97–317) still interacted with HNF4A (Fig. 6B). In addition the C-terminal region does not appear to be involved as a construct spanning AA318–372 did not interact with HNF4A; this was confirmed by GST pull-down where region 1–240 was sufficient for binding.

The interaction picture is however complex. First of all, multiple epitopes are involved as we noted that the C-terminal half (241–372 and 162–372) could weakly interact with HNF4A (Fig. 6B and C). Furthermore the interaction was enhanced by removing the C-terminus (1–240 vs. Wt), suggesting that in the intact protein the C-terminal region of TRIB1 plays a dominant role in reducing net binding, perhaps by masking a high affinity binding site and providing an alternate, weaker binding site. Refining the interaction requirements narrowed down an interaction epitope in the N-terminus but also demonstrated that region 97–372 could interact with HNF4A, confirming that presence of at least two distinct interfaces (Fig. 6B **)**. Finally, pull-down assays performed on HepG2 nuclear lysates confirmed that a fragment spanning AA1–97 was sufficient for HNF4A binding (Suppl Fig. 7).

### Mapping of the HNF4A region interacting with TRIB1

**Figure 7:**
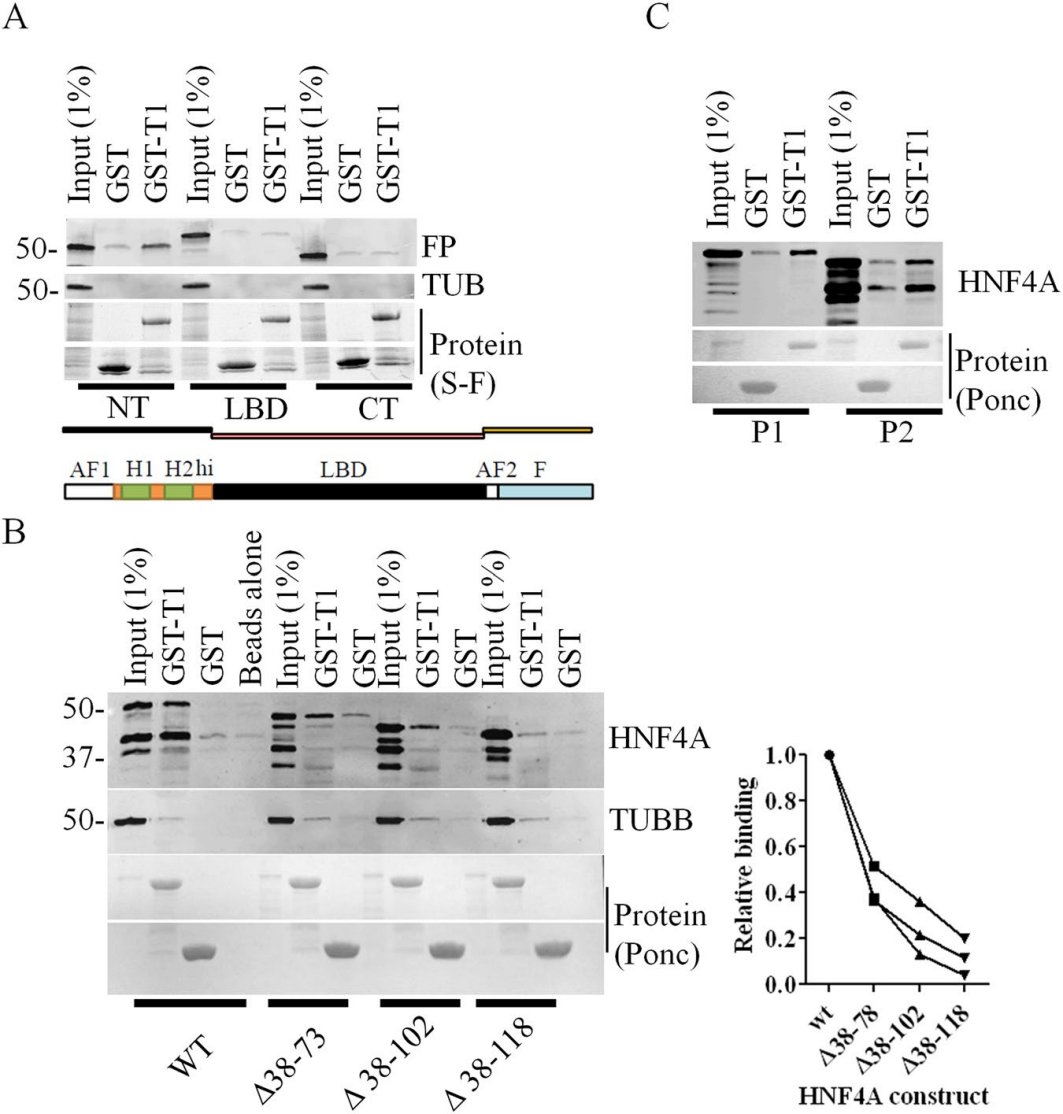
HNF4A interacts with TRIB1 via its N-terminus. Fragments (**A**) or deletions (**B**) of the HNF4A P2 variant or the full-length P1 or P2 variants of HNF4A (**C**) were expressed in HEK293T cells and subjected to pull-downs using either GST or GST-TRIB1 (GST-T1). Results are representative of three independent pull-down assays. In B, quantification of three distinct pull-downs (±S.D) is shown; quantification was performed using the high molecular band of each form and is expressed relative to Wt binding (see Methods). Total protein was resolved on a 4–15% gradient (**A**) or a 10% SDS-PAGE gels (**B,C**). Proteins were imaged using either Stain-Free methodology (“S-F”, A) or Ponceau (“Ponc”, B and C).

Earlier pull-downs indicated that transfected full-length HNF4A resulted in two distinct bands migrating as ~48 kDa and ~42 kDa that were systematically co-isolated by TRIB1 pull-downs. While the slower form was consistent with its predicted mass, we hypothesized that the second form may stem from an alternative translation initiation event. This hypothesis was based on the observation that 1) the epitope recognized by the antibody is located at the very C-terminus of HNF4A, narrowing down mass differences to the N-terminus and 2) initiation at the next methionine (Met84) is predicted to yield a product of ~42 kDa. Indeed, when HNF4A was expressed as three distinct fragments spanning the entire protein, fused to CFP, only the N-terminus (including part of the hinge region) displayed affinity for TRIB1 (Fig. 7A). Further deletions within the N-terminal fragment revealed a progressive loss of interaction (Fig. 7B). Thus the entire N-terminal region of HNF4A is required to maximize binding.

Several isoforms of HNF4A, which result from alternative splicing/promoter usage and reflect tissue specific functions, have been reported. For our original mapping efforts, the shorter P2 form of HNF4A (corresponding to HNF4α7) was chosen to simplify mapping, under the assumption that, by similarity with RAR, HNF4A interacted with TRIB1 via its activation domains. The two HFN4A isoforms share most of their sequence with the exception of the N-termini. In view of the contribution of the HNF4A N-terminal region for its interaction with TRIB1, its relative affinity for both forms (HNF4α7 and α2) was tested in pull-down assays. When expressed in 293 T cells both forms bound TRIB1 (Fig. 7C).

### CEBPA and HNF4 interact with TRIB1 via distinct epitopes

Finally we asked whether TRIB1 displayed exclusive or additive interactions with CEBPA and HNF4A. The evidence of multiple interacting regions on TRIB1 would suggest a possible non-exclusive binding model whereby TRIB1 could interact with both proteins simultaneously. First the ability of TRIB1 to interact with CEBPA was confirmed (Suppl Fig. 8). Interestingly HNF4A and CEBPA exhibited similar binding affinities to TRIB1 as expressed relative to input signal.

**Figure 8:**
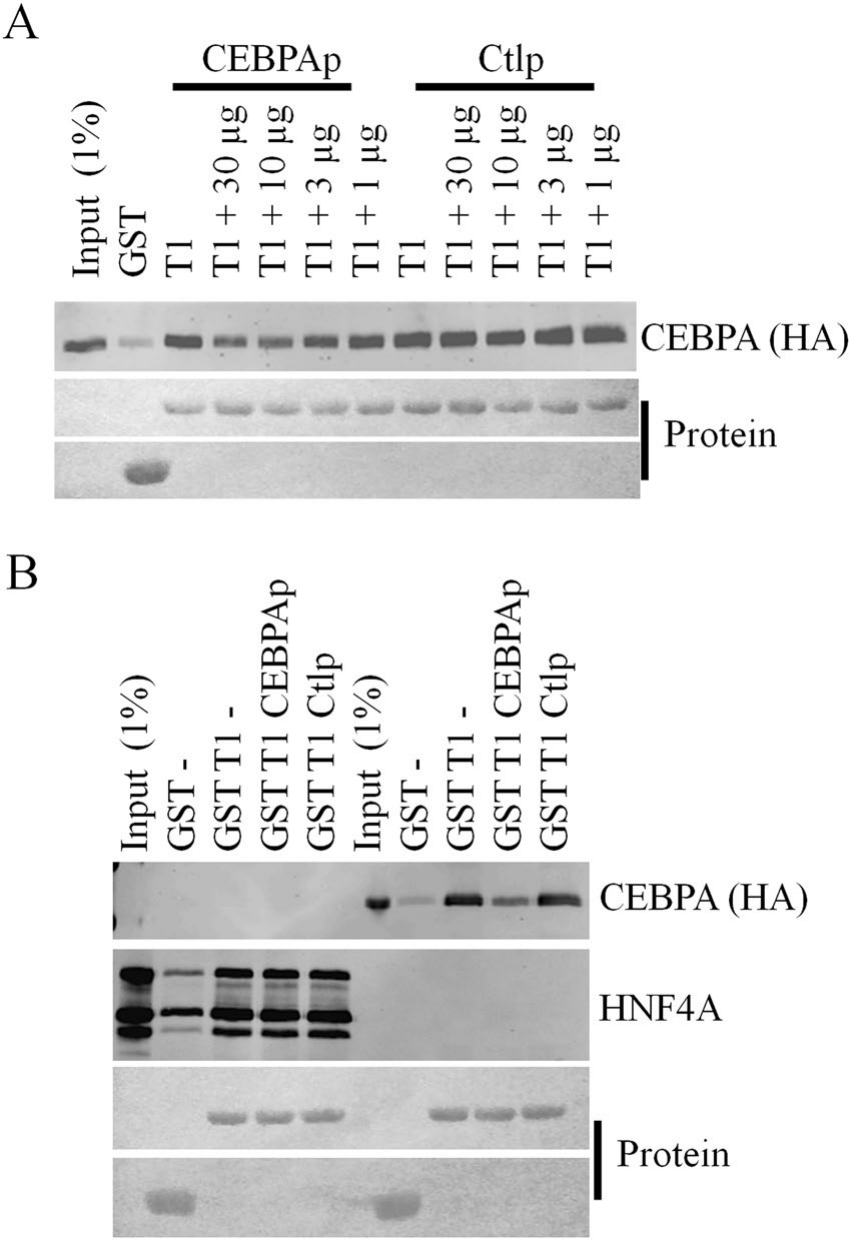
A CEBPA derived peptide interferes with CEBPA binding, but not HNF4A binding, to GST-TRIB1. Pull-down assays of CEBPA-HA and HNF4A. **A**, lysates of HEK293T transfected with plasmids coding for CEBPA-HA were incubated with GST-TRIB1 or GST in the presence of increasing concentrations of either a peptide matching the CEBPA interface (CEBPAp) or a control (Ctlp), substituted peptide. CEBPA in isolates was quantified by Western blotting and protein input by Ponceau. **B**, as in A except that lysates expressing either HNF4A or CEBPA were used in each pull-down assay. Where indicated, 10 µg of either peptide were used. Experiments were performed twice with comparable results.

To probe the interfaces involved, competition assays employing peptides derived from CEBPA were optimized. This approach took advantage of the recent identification of a region of CEBPA sufficient for binding TRIB1^27^. To overcome solubility limitations, targeted non-essential point mutations were introduced first and the resulting peptide, together with a control peptide containing key mutations at conserved residues, were tested for their ability to prevent TRIB1/CEBPA interaction. Inclusion of the CEBPA-derived peptide, but not the control, reduced co-isolated CEBPA (Fig. 8A). However, when tested for their impact on HNF4A, neither peptide affected HNF4A binding with TRIB1, consistent with the utilization of distinct TRIB1 interfaces (Fig. 8B).

## Discussion

Here we have explored the functional relationship between *HNF4A* and the *TRIBBLES*, focusing on TRIB1. Our previous work revealed that *HNF4A* suppression led to *TRIB1* upregulation and that *TRIB1* suppression using two distinct approaches (siRNA and ASO) targeting different regions of the TRIB1 transcript reduced HNF4A levels and function^11^. This suggested the presence of a regulatory network involving both proteins. We now demonstrate that *TRIB1* suppression and *TRIB1* overexpression respectively reduce and increase HNF4A activity. In addition we demonstrate that the other two mammalian *TRIBBLES* are also required for optimal HNF4A protein expression, although their contributions are more limited in that regard. Although off-target effects remain possible and should be excluded by using additional siRNA targeting distinct regions of *TRIBBLES* transcripts, the finding that all three *TRIBBLES* siRNA reduce HNF4A protein expression reinforce the notion that individual *TRIBBLES* are contributing to maintain optimal HNF4A levels.

In addition to demonstrating a requirement for the *TRIBBLES*, our results reveal that the three TRIBBLES can physically associate with HNF4A *in vitro*; further work will be needed to assess whether TRIB2 and TRIB3 can directly interact with HNF4A *in vivo*. While we demonstrate the presence of an *in vivo* complex between (overexpressed) TRIB1 and HNF4A, contributions of this complex to HNF4A function remain unclear. Judging from the low abundance of TRIB1 relative to HNF4A, inferred from both qRT-PCR (~60 X difference) and Western blot evidence, the endogenous TRIB1 is predicted to sequester only a small fraction of the HNF4A population at any given time. Thus TRIB1, and perhaps all the TRIBBLES, operate in part by steering subpopulations of HNF4A towards particular transcriptional programs. HNF4A is unique among the nuclear factor superfamily in that coactivator binding, rather than ligand binding (which is necessary but not sufficient) locks its active conformation^28^. Binding of TRIB1 to its N-terminal, which includes its DNA binding domain and part of the hinge region linking it to the LBD, is predicted to affect its ability to interact with cognate DNA sequences. As *TRIB1* overexpression was associated with increased transcriptional output on a reporter system, anchoring of TRIB1 on HNF4A is predicted to augment its activity. Alternatively, cellular changes secondary to TRIB1 overexpression may be invoked. Nonetheless, binding to the N-terminal region of TRIB1 leaves open the possibility for the formation of higher order complexes comprised of HNF4A and other interactors identified over the years, which have been shown to bind elsewhere on TRIB1 (e.g. CEBPA, CEBPB, MEK1, MLXIPL, RARA, RXRA, SAP18). TRIB1 (and the TRIBBLES in general) may thus serve as a transcription modulator that operates by integrating converging transcription programs.

HNF4A stands out amongst the TRIB1 interactors given that its expression correlates positively with that of *TRIB1*. This may reflect in part distinct binding modalities. The observation that binding of HNF4A to TRIB1 resists competition with the CEBPA peptide is consistent with this model. Thus binding by HNF4A does not, unlike CEBPA association, lead to its degradation. Unfortunately, limited additional information can be gleaned from the relatively better characterized CEBPA/TRIB1 interaction picture as it still unclear where CEBPA docks on the TRIB1 surface. Some binding characteristics have been defined however: a requirement for the pseudokinase domain, an intact pseudocatalytic loop as well as the MEK binding interface (but not the more distal COP1 binding region)^27, 29^. Interestingly we observed a reduction in binding when the MEK binding region was deleted. As the C-terminal tail is not needed for binding, we speculate that the deletion may lead to its inappropriate positioning over the interaction interface located close to the N terminus, which accords with the ability of the C-terminal tail to fold back onto the N-terminus of the protein^27^.

Our findings are consistent with a model (Suppl Fig. 9) whereby TRIB1 is required to maintain a suitable cellular environment for proper HNF4A expression and function. In the absence of *TRIB1* (and *TRIBBLES*), ensuing changes lead to reduced HNF4A levels. How this is achieved is likely multi-faceted as HNF4A was reduced either in the absence of significant transcript change (HepG2) or its presence (HuH-7 and primary human hepatocytes)^11^. As *TRIB1* overexpression did not measurably increase HNF4A protein level, this further suggests that its impact on HNF4A stability is indirect or requires limiting factors that remain to be identified. Judging from the profound impact of silencing of *TRIB1* in various liver models, numerous pathways could be affected. One of these may involve the MEKs which orchestrate a plethora of signaling cascades and which constitute *TRIBBLES* regulatory targets. The TRIBBLES exhibit a complex regulatory relationship with the MAPKKs^30, 31^ and their absence would be predicted to influence MAPKK activity, in turn affecting HNF4A. Indeed, activation of MEK1/2 by IL1β has been reported to attenuate HNF4A levels, by reducing transcript level as well as promoting protein degradation^32^. In addition, HNF4A has been reported to be destabilized by successive SUMOylation and ubiquitination events during hepatocyte maturation^33^. By interfering with these processes, TRIB1 could be instrumental in helping to sustain higher basal HNF4A levels.

There is now considerable evidence pointing to a convergence of HNF4A and TRIB1 in regulating energy metabolism. Both proteins have been linked to glucose metabolism in the liver. HNF4A is required for gluconeogenesis, a role it performs in conjunction with the PPAR co-activator PPARGC1A (PGC-1alpha)^34^. Recent work has uncovered a role for TRIB1 in the regulation of circulating glucose levels in the mouse^29^. Moreover *TRIB1* suppression is associated with the widespread downregulation of enzymes involved in glucose metabolism in human primary hepatocytes^11^. *HNF4A* and *TRIB1* have also been associated with altered lipid traits in humans^35, 36^. A large GWAS identified a missense mutation in *HNF4A* that correlates with reduced HDL levels^35^ and common non-coding variants within the *HNF4A* gene have been linked to elevated plasma lipids^37^. *TRIB1* polymorphisms located ~30 kb away have been linked to changes in plasma triglycerides, LDL-cholesterol and HDL-cholesterol^7, 38–40^. A direct contribution of TRIB1 is also supported by animal studies demonstrating that circulating plasma lipids and *Trib1* levels are inversely correlated^10, 29, 41, 42^. This effect may be due in part due to its effect on *MTTP* encoding the microsomal triglyceride transfer protein, which is under the transcriptional control of HNF4A and is essential for VLDL lipidation and secretion^43^. Indeed, *MTTP* expression is proportional to that of *TRIB1* ^44^. Mice lacking hepatic *Hnf4a* show a dramatic reduction in *Mttp* expression and exhibit severe reductions in circulating cholesterol and triglyceride levels, and hepatic lipid accumulation (hepatosteatosis)^18^. Significantly, *TRIB1*-linked SNPs are associated with nonalcoholic fatty liver disease (NAFLD), defined as hepatosteatosis^29^. This phenotype is also observed in liver-specific *Trib1* knockout mice and cannot be phenocopied by *CEBPA* overexpression, consistent with a role for TRIB1 in orchestrating non-CEBPA related functions^10^. Here we establish that the *TRIBBLES*, and *TRIB1* in particular are positive regulators of *HNF4A*, supporting the hypothesis that reduced HNF4A, may in part account for the CEBPA-independent effects of *TRIB1* suppression.

## Methods

### Cell culture and treatments

HepG2 and HEK293T cells were obtained from ATCC (www.atcc.org) and maintained in low or high glucose DMEM (LG-DMEM/HG-DMEM), respectively. HuH-7 is a human differentiated hepatocellular carcinoma (JCRB cell bank). SiRNA treatments were performed for 48–72 h at 20 nM, using 2 ul of RNAiMax per 12 well plate well; siRNAs are listed in the Supplementary Materials section.

### Quantitative Real-Time RT-PCR and qPCR arrays

RNA was isolated using a High Pure RNA isolation kit (Roche) and reverse transcribed. Quantitative PCR was performed on a LightCycler480 using the Roche LightCycler480 SybrGreen I Master mix (Roche). Total RNA (0.5–1 μg) was reverse-transcribed with the Transcriptor First Strand cDNA synthesis kit (Roche Diagnostics) using a combination of oligo-dT and random hexamers. Crossing point (Cp) values for each sample were first normalized to *PPIA*. Oligonucleotides used for Quantitative Real-Time RT-PCR (qRT-PCR) are listed in the Supplementary Materials section.

### Expression constructs

Human *TRIB1* (372 aa) was subcloned using PCR and restriction digests from Origene’s construct (PCMV6-XL5TRIB1). *TRIB1* was cloned in pLVX (HepG2 and HuH-7 stables) or PLVXHIS (in frame with a C-terminal HIS tag), pCMV-Tag (transfection experiments) or pGEX4 T (bacterial expression). *TRIB3* was amplified from liver cDNA using Q5 polymerase. The P2 form of *HNF4A* (*α7;* NP_001025174*)*) and *HNF1A* were amplified from human liver cDNA and cloned in pLVX. *HNF4A* (*α2*; NP_000448), *TRIB2* and *CEBPA* constructs were purchased from Bio Basic Inc. The CEBPA coding sequence, corresponding to the p42 form (Uniprot identifier P49715-2) was optimized to reduce its GC content using IDT’s (http://www.idtdna.com/site) proprietary tool. All three constructs contained C-terminal spacers and HA tags. PCR and Mutagenesis were performed using the Q5 high fidelity polymerase as per the supplier’s (New England Biolab) instructions. For the BirA system, the mycBioID plasmid was a gift from Kyle Roux obtained via Addgene (Addgene plasmid 35700). *TRIB1* was cloned in C-terminal to the mycBioID tag using conventional molecular biology methods. Integrity of the constructs was confirmed by sequencing of the open reading frames.

### Protein interaction studies

Recombinant proteins were isolated 48 h after transfections with either Fugene 6 (Promega) or Lipfectamine 3000 (ThermoFisher) and the appropriate expression constructs. Cells were rinsed in PBS, scraped and lysed for 5 min in ice cold IP buffer (20 mM HEPES, 120 mM NaCl, 0.5% NP40, pH 7.4) containing phosphatase and protease inhibitors cocktails (Roche Life Science). Insoluble debris were cleared by centrifugation (1 min, 15,000 x *g*) and complexes were snap-frozen to be used as a source of HNF4A for pull-down experiments or used fresh for coIP experiments. For bacterial expression (Rosetta2 strains, Novagen), fusion proteins were cloned in pGEX4T1. Overnight bacterial cultures were diluted 1:100 in 100 ml fresh LB broth (+Ampicillin) and bacteria were grown until OD600 of ~0.7 and induced for 3 h with 0.2 mM isopropyl beta-D-thiogalactoside at 30 °C. Bacteria were recovered in 5 ml of 25 mM Tris-HCl, 0.15 M NaCl, 1% Triton X-100, 0.5 mM EDTA, pH 8 and sonicated in a Bioruptor (Diagenode). Bacterial debris were removed by centrifugation and fusion proteins were bound to GST agarose beads (GE Health Care Life Sciences) and washed in IP buffer. Pull-downs were then performed using 0.2 mg of HEK293T lysates transfected with either HNF4A or CEBPA (snap frozen and stored at −80 °C) or HuH-7 lysed in 0.5 ml of IP buffer and 10 µl of GST bead volume. For pull-down assays, prey binding was first corrected for the amount of bait and then divided by input intensity; binding was then normalized to the wild-type form.

Peptide competition assays were performed using either a CEBPA derived peptide (Biotin-SEICEDENSIDISAYIDPAAFND) with conservative point mutations to facilitate synthesis, or a control peptide harboring damaging mutations (Biotin-GGACEHATSDDASAYADPAAFND) (Novopep, Shanghai, China). Peptides were added concurrently with the prey lysates.

For BioID tagging, 6 well plates of HEK293T were co-transfected with 2 µg per well with HNF4A (1.5 µg per well) and either empty plasmid (0.5 µg), BirA (0.1 µg) or BirATRIB1 (0.5 µg) for 48 h. During the last 24 h of transfection, medium was replaced with fresh medium containing biotin (0.1 mM). Cells were quickly rinsed and recovered in ice cold PBS, resuspended briefly and hot (95 °C) lysis buffer (50 mM Tris, pH 7.4, 200 mM NaCl, 1% SDS, 5 mM EDTA, 1 mM DTT) was added to the samples. Lysates were mixed well and transferred to 95 °C for a further 10 min with occasional mixing. Samples were then diluted 5 X in dilution buffer (50 mM Tris, pH 7.4, 100 mM NaCl, 0.25% TRITON X-100, 5 mM EDTA) and sonicated 2 X 5 min (30 /30 cycles) in a Bioruptor (Diagenode). Insoluble material was removed by centrifugation at 15,000 x *g* for 10 min and the supernatant was incubated with Streptavidin-coupled magnetic beads (Life Technologies) for 1 h. Samples were washed 4 X in dilution buffer and analyzed by Western blot. Streptavidin (IRdye800CW; LI-COR) diluted at 1:50,000 in PBS was used to detect the biotin-modified protein.

### Western blotting, immunocytochemistry and immunoprecipitation

Cellular extracts were obtained by the addition of RIPA buffer (50 mM Tris-HCl, 1% NP40, 0.1% SDS, 0.5% NaDeoxycholate, 0.15 M NaCl, pH 8.0) supplemented with Complete protease inhibitors and PhoStop tablets (Roche) for 5 min to the permeabilized cells on ice. Western blotting was performed on ~30 µg per sample or isolate, as appropriate,that was resolved under reducing and denaturing conditions on TGX 4–15% polyacrylamide gels (Bio-Rad). Where indicated Stain-Free pre-cast gels (Bio-Rad) were used and imaged for total protein content prior to transfer using tryptophan-trihalo fluorescence. Proteins were transferred to nitrocellulose, blocked in PBS/5% milk or Odyssey Blocking buffer (Li-Cor) for 20 min prior to detection. Detection was performed for 16 h using 1:2000 dilutions of the primary antibodies and cognate LI-COR secondary antibodies (1:25,000 dilutions in PBS). All washes were in PBS (4 × 5 min). Imaging was performed on an Odyssey Imager (LI-COR).

For immunocytochemistry, cells seeded on glass coverslips were transfected for 48 h with HNF4A and/or TRIB1 constructs and fixed in 4% PFA for 15 min. Permeabilization was achieved with 0.1% Triton X-100 in PBS for 10 min and followed by a blocking step in 5% bovine serum/PBS (10 min) and incubation for 1 h with antibodies diluted at 1:500 in PBS. Alexa-coupled secondary antibodies were used (Donkey anti-Goat 633 and Donkey anti-Rabbit 546; Life Technologies) at 1:2,000 dilution. Image acquisition was performed using an Olympus confocal microscope equipped with a 100 X oil immersion lens and operated by the FluoView software. Image processing was performed using the FluoView viewer software with subsequent minor adjustments in Microsoft PowerPoint. Antibodies used are listed in the Supplementary Materials section.

Immunoprecipitation of TRIB1 was performed on denatured lysates. HuH-7 cells were treated for 48 h with siRNAs, washed in PBS, harvested by scraping and lysed in 1 ml (per two 10 cm dishes) of PBS/0.1% Triton X-100 supplemented with Complete protease inhibitors (Roche) for 5 min with rotation; using flagTRIB1 as a guide, optimization indicated that this condition was sufficient to recover significant (~10% of total) TRIB1. Samples were then centrifuged and the soluble fractions were recovered, adjusted to 1% SDS and heated at 95 °C for 5 min. Following a dilution in four volumes of 25 mM Tris-HCl, 0.15 M NaCl, 1% Triton X-100, 0.5 mM EDTA, pH 8 to displace the SDS from the protein surface and allow the formation of antigen-antibody complexes^45^, the lysates were cleared through 0.4 µM PVDF disks. The filtrates were then subjected to immunoprecipitation for 16 h at 4 °C using 10 µl of a rabbit antibody to TRIB1 (N2C3, Genetex) and 20 µl of rinsed protein A/G mix (PureProteome Magnetic Beads, Millipore). Detection was performed using a goat polyclonal antibody to TRIB1 (Genetex).

### Statistical analyses

To assess statistical significance of changes relative to a control value (e.g. Non-Target oligonucleotide), unpaired 2-tailed student’s t-tests were performed unless mentioned otherwise. For in-between group comparisons, one way ANOVA was performed, followed by Tukey’s post-hoc test in GraphPad Prism 5.

## Electronic supplementary material

Supplementary Figures and Materials
