# Supplementary material for: TRIB1 is a positive regulator of hepatocyte nuclear factor 4-alpha
Source: Sci Rep. 2017 Jul 17;7:5574. doi: 10.1038/s41598-017-05768-1 (PMC5514136; doi:10.1038/s41598-017-05768-1)
Supplement: Supplementary file 1 — Supplementary Figures and Materials [file 41598_2017_5768_MOESM1_ESM.pdf]

Supplementary figures and information

TRIB1 is a positive regulator of hepatocyte nuclear factor 4-alpha

Sébastien Soubeyrand, Amy Martinuk and Ruth McPherson

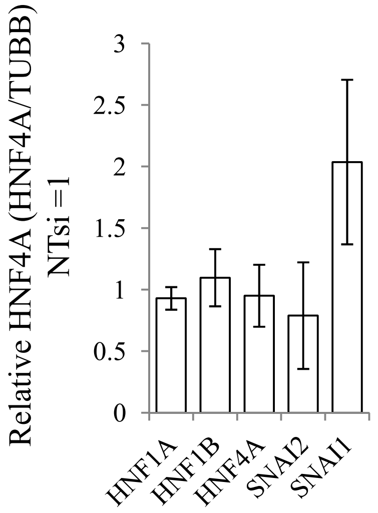

Supplementary Figure 1: *TRIB1* suppression in HepG2 cells leads to impaired HNF4A signaling but no changes in HNF4A transcript level. Indicated transcripts were measured from RNA isolated from HepG2 transfected with a *TRIB1* antisense oligonucleotides for 48 h. Data represent the average of 4 experiments ± 95% CI. Only *SNAI1* was significantly changed ($p < 0.05$ vs Non-target control, Student's 2-tailed t-test)

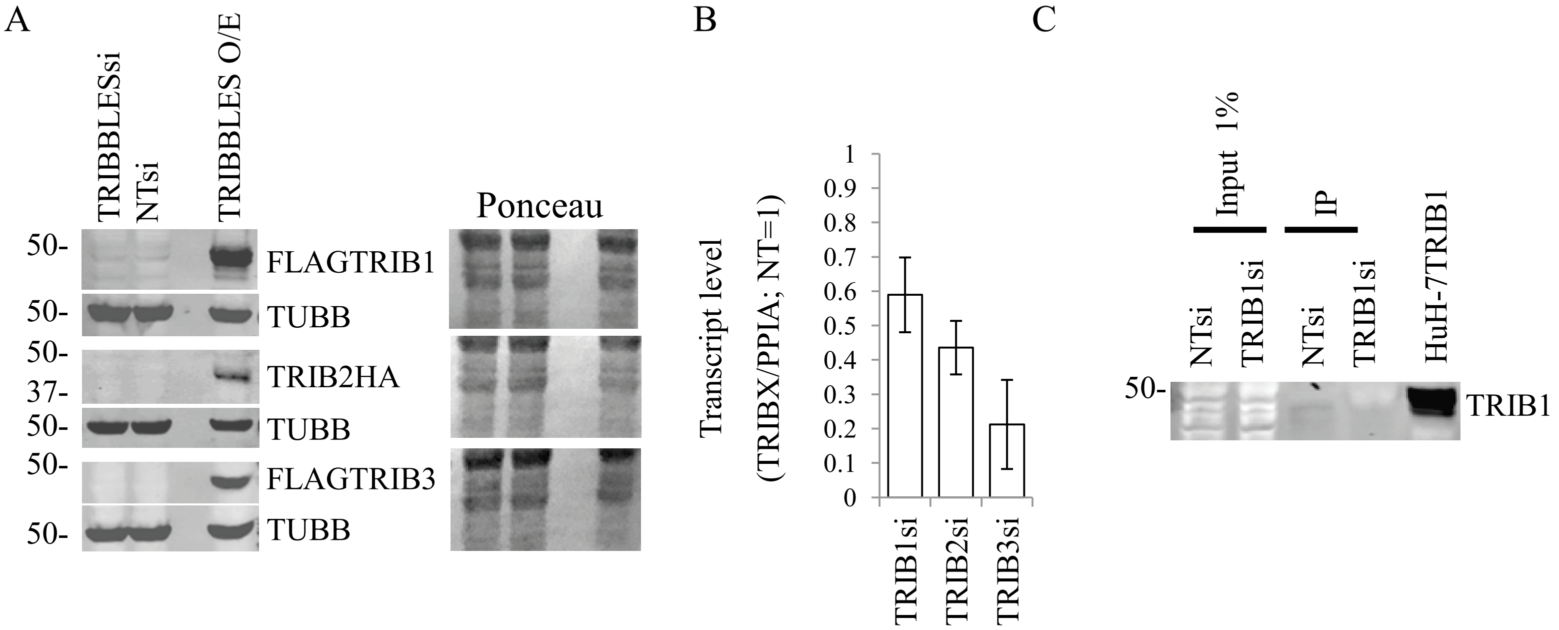

Supplementary Figure 2: *TRIBBLES* siRNA controls. A, impact of *TRIBBLES* silencing on TRIBBLES protein expression assessed by Western blot (8% SDS-PAGE gels). Samples of HuH-7 cells transfected for 24 h with individual *TRIBBLES* expressing plasmids (FLAG or HA tagged as indicated; 1 µg of plasmid and 3 µl of lipofectamine 3000 per 400 mm^2) were included as positive controls. Detection was performed using TRIB1, 2 or 3 as well as β-Tubulin (TUBB) antibodies, as appropriate. Middle, corresponding Ponceau showing the 35 to 50 kDa range where the TRIBBLES migrate. B,validation of the knock-down for each cognate *TRIBBLES* siRNA assessed by qRT-PCR quantification; all changes are significantly different from NT (1-tailed Student's t-test, $p< 0.05$). C, Western blot of TRIB1 immunoprecipitated from HuH-7 cells treated with NTsi or TRIB1si. Immunoprecipitation of endogenous TRIB1 from HuH-7. Immunoprecipitation was performed on 5 mg of HuH-7 extracts using a cognate rabbit antibody and detection was performed with a goat anti-TRIB1. A lysate (5 µg) of HuH-7 cells stably expressing recombinant TRIB1 is included as a positive control. The major endogenous TRIB1 band migrated as a protein of mass 47 kDa, larger than its theoretical mass of 41 kDa but in line with recombinant TRIB1's mass. The white background above the TRIB1 signal in the IP samples is likely due to the presence of the heavy chains of the rabbit antibody used for immunoprecipitation.

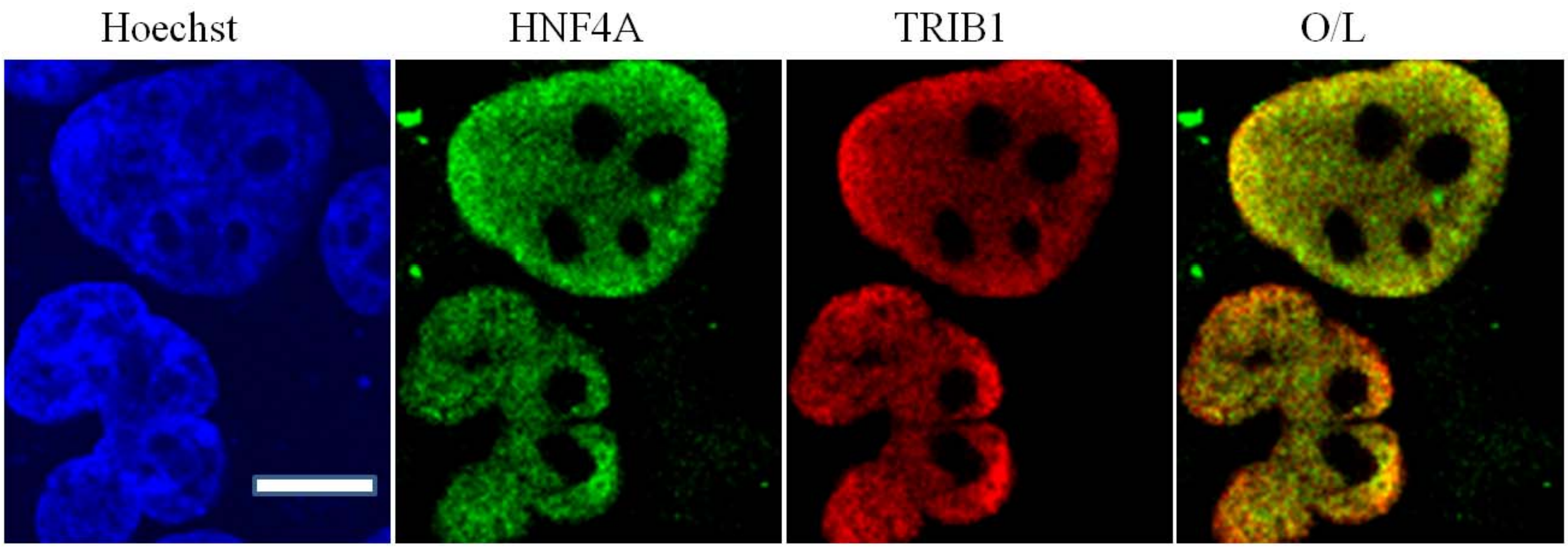

Supplementary Figure 3: Partial overlap of TRIB1 and HNF4A signals in transfected HEK293T cells. HNF4A and flagTRIB1 expression plasmids were transfected in HEK293T for 24 h, fixed and probed for HNF4A (green) and TRIB1 (red) by immunocytochemistry. Scale bar: 10 µM. Yellow signals indicate populations which overlap within the resolution limit (~ 200-300 nm) of the method.

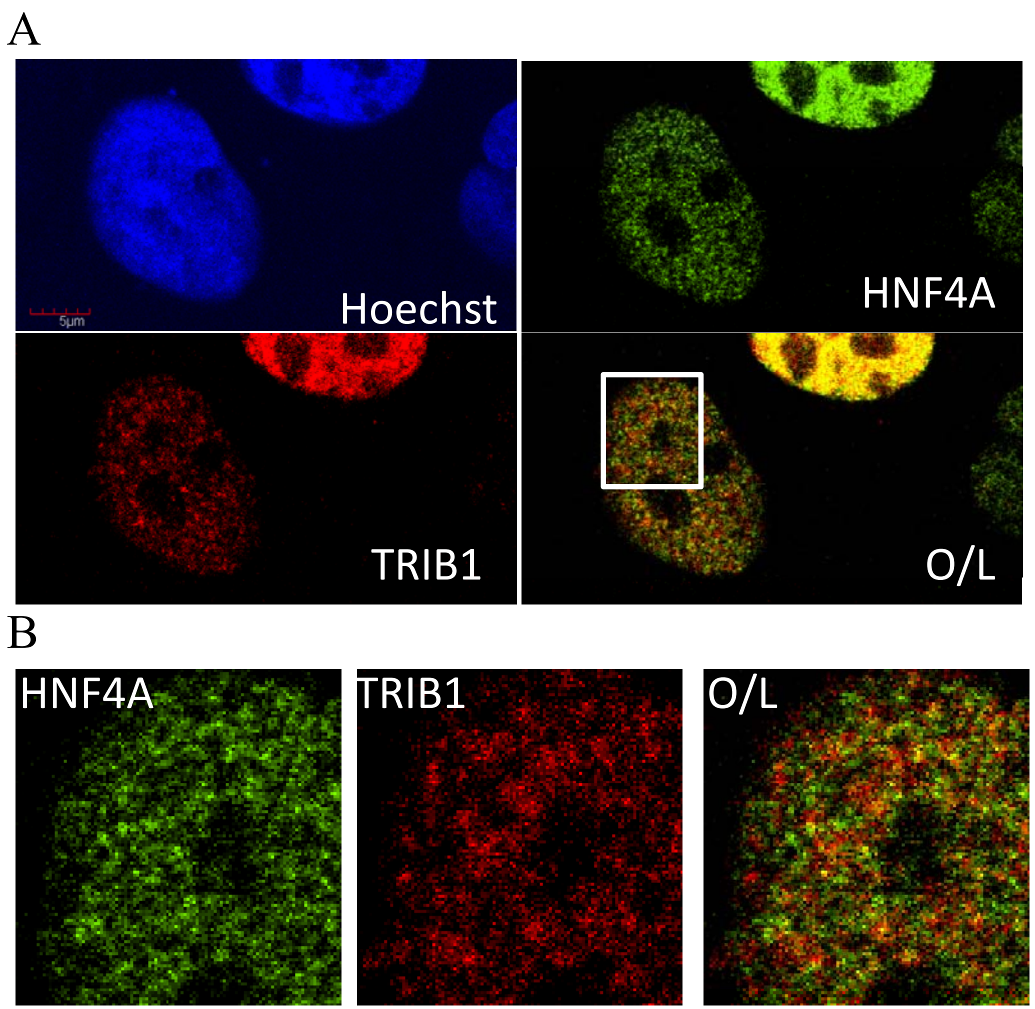

Supplementary Figure 4: Partial overlap of TRIB1 and HNF4A signals. A pool of HuH-7 cells stably transduced with TRIB1 was fixed and examined for HNF4A (green) and TRIB1 (red) by immunocytochemistry. White rectangle highlights a region which is further magnified in B.

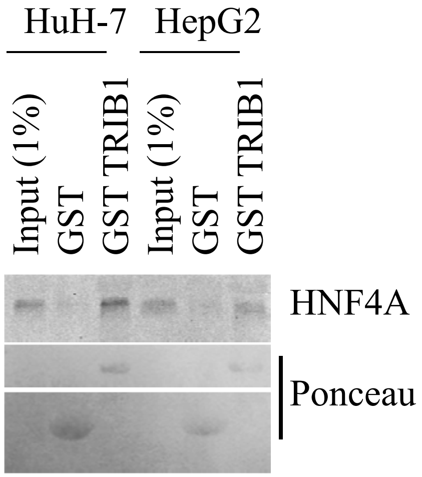

Supplemental Figure 5: TRIB1 interacts with endogenous HNF4A (HepG2 and HuH-7). Nuclear lysates from HepG2 and HuH-7 cells were subjected to pull-down assays using GST or GST TRIB1, as indicated. Bound material was washed and analyzed by Western blotting using a HNF4A-specific antibody. A representative blot is presented with a matching Ponceau stain that shows the relative amount of bait used.

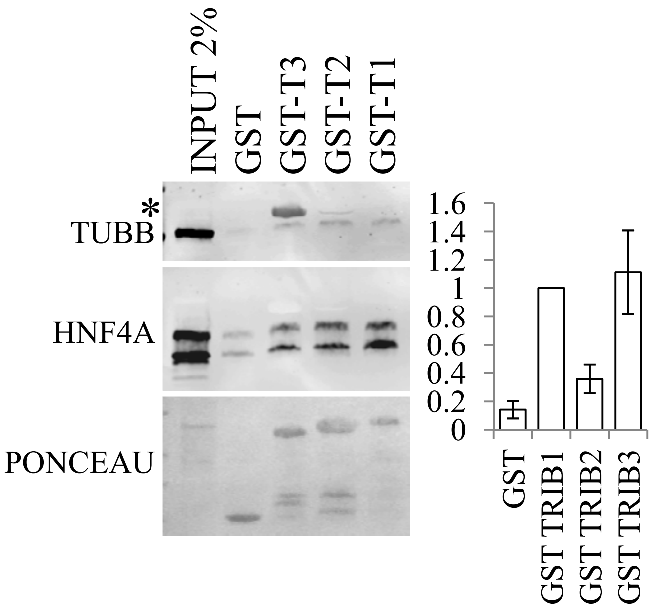

Supplementary Figure 6: All TRIBBLES interact with HNF4A. Pull-downs were performed on 293T lysates transfected for 24 h with *HNF4A*. * indicates a cross-reactivity of the TUBB and/or secondary antibody with the GST-T3 fusion protein. Quantification of 3-4 bindings (± S.D.), corrected for bait amount and normalized to GST-TRIB1, is shown on the right. GST and GST-TRIB2 were significantly different from each other ($p = 0.01$; Student's 2 tailed t-test). Both bound significantly less ($P < 0.01$, ANOVA, Tukey's post test) relative to GST-TRIB1 and -TRIB3.

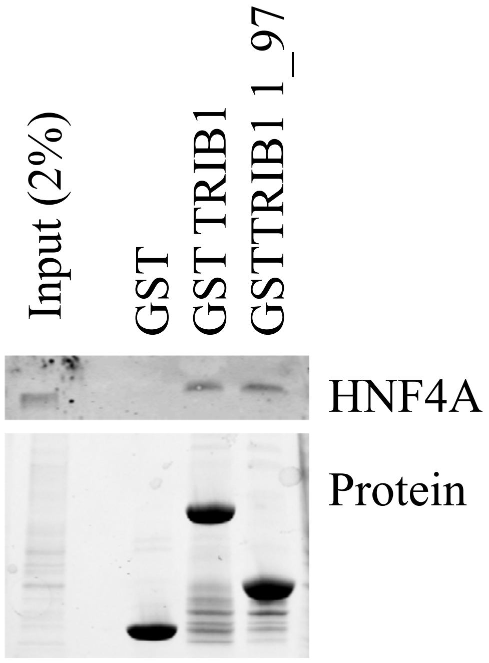

Supplementary figure 7: TRIB1 interacts with endogenous HNF4A via its N-terminus. HepG2 nuclear extracts were subjected to pull-down experiments using GST-TRIB1 and GST-TRIB1_1-97. Top, western blot probing for HNF4A and bottom, matching protein (Stain-free gel) is shown.

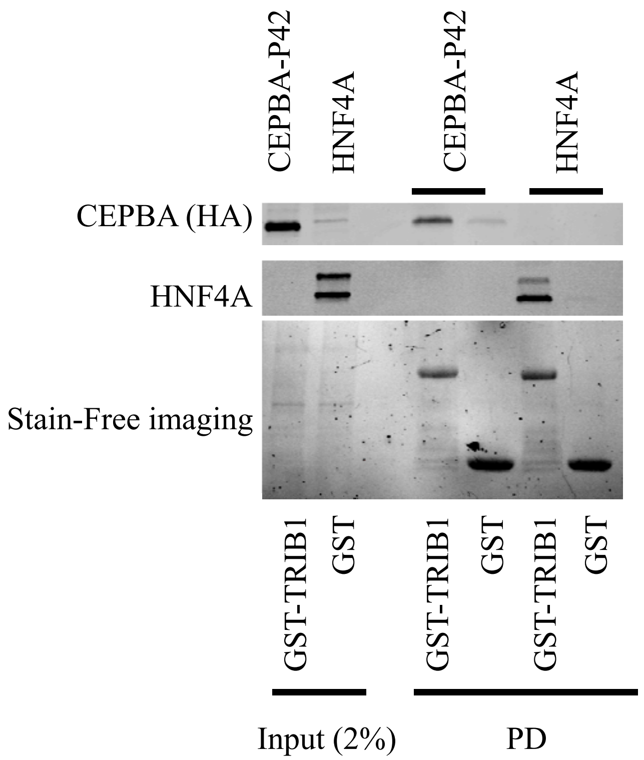

Supplementary Fig 8: Comparable binding of CEBPA and HNF4A to TRIB1. HEK293T cells were transfected with plasmids encoding HNF4A (P2, α7 form) or C-terminally tagged HA-CEBPA (p42 form). Cell lysates were then subjected to pull-downs with the indicated baits, resolved on a 4-15% gradient gel (Stain-Free) and analyzed by Western blotting.

.

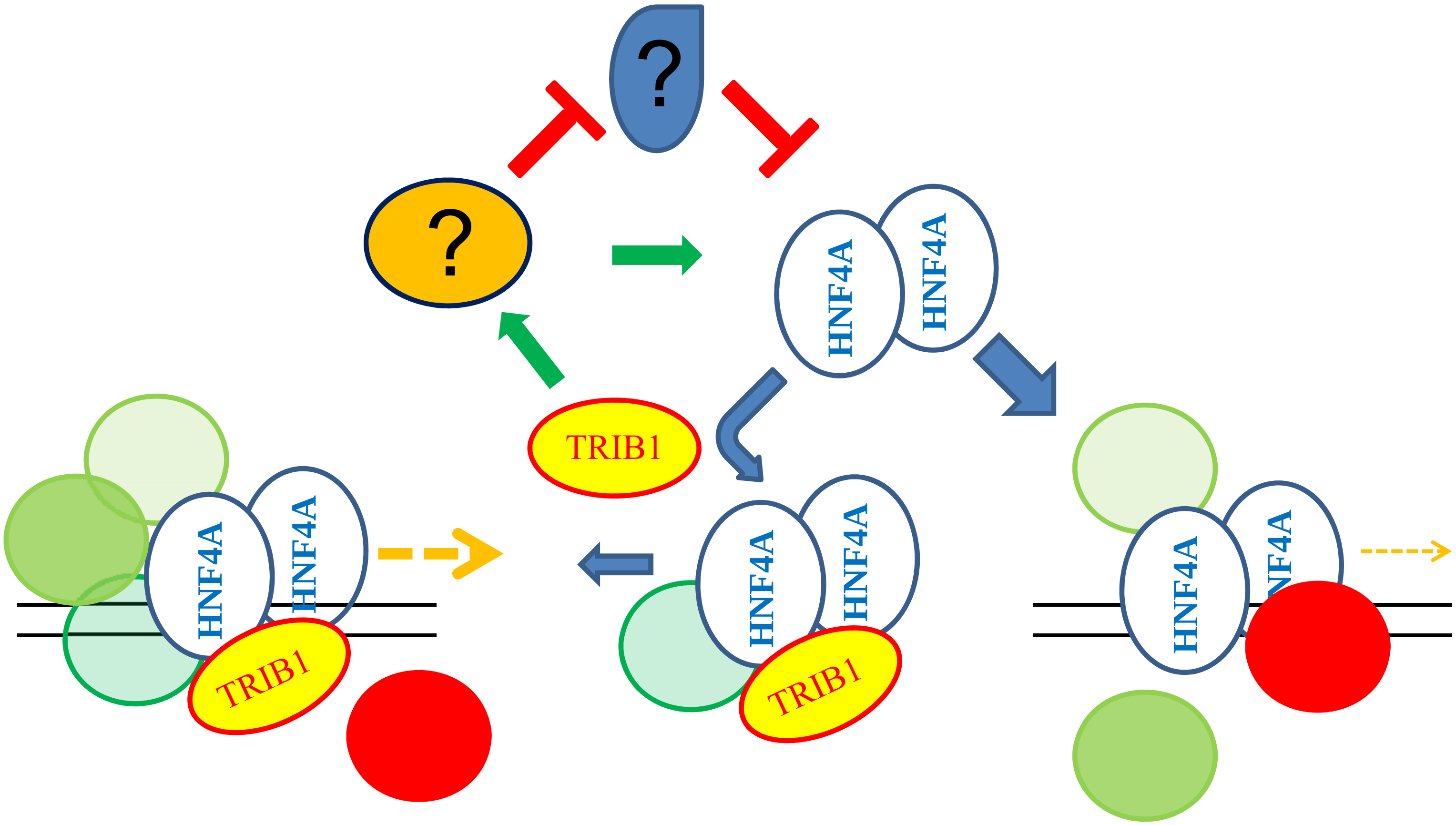

Supplementary Fig 9: Regulation of HNF4A by TRIB1 occurs at multiple levels. Model of the interplay between HNF4A and TRIB1 in hepatocytes. Presence of TRIB1 is necessary to maintain homeostasis and sustain HNF4A levels, through transcriptional and post-transcriptional mechanisms. Only post-transcriptional regulatory events are illustrated. Green arrows and red Ts represent stimulatory and inhibitory intermediaries that remain to be identified. In addition TRIB1 can interact via at least 2 distinct regions with a subset of the HNF4A population directly which may permit its asymmetric association with the HNF4A homodimer. The interaction is DNA-independent but predicted to impact DNA-bound HNF4A and alter its ability to effect transcription. For instance, binding of TRIB1 to HNF4A may prevent recruitment of transcription repressors (red sphere) and/or facilitate its interaction with co-activators (green spheres). These interactions ultimately converge in ensuring optimal HNF4A function.

Antibodies

CEBPA: D56F10 (#8178 Cell Signaling Technology)

FLAG: M2 (Sigma-Aldrich)

GFP: G1544 (Sigma-Aldrich)

HA: 6E2 (#2367 Cell Signaling Technology)

HNF4A: C11F12 (Cell Signaling Technology)

HNF1A: #12425 (Cell Signaling Technology)

MTTP: N-17 (SC-33116, Santa Cruz Biotechnology Inc)

Myc: 9B11(#2276 Cell Signaling Technology)

TRIB1 (immunoprecipitation): GTX111960 (GeneTex)

TRIB1 (Western blot):GTX88755 (GeneTex)

TRIB2: F-5 (SC-376776, Santa Cruz Biotechnology Inc)

TRIB3: G-10 (SC-271572, Santa Cruz Biotechnology Inc)

TUBB: GTX11307 (GeneTex)

Oligonucleotides

siRNAs

All siRNA were obtained from Life Technologies (Ambion):

control siRNA (NT #1)

CEBPA siRNA (S2889)

HNF4A siRNA (S6698)

TRIB1 siRNA (silencer select, UCUGUUGGGAUGAGUGACU)

TRIB2 siRNA (S26280)

TRIB3 siRNA (S33710)

qRT-PCR oligos

TRIB1

TTCAAGCAGATTGTCTCCGC

AGTGGTGTTGAGGATCTCAG

TRIB2

TGCGTTTCTTGTATCGGGAAATAC

CATAGCTTCGCTCAAAGAACAC

TRIB3

CTGCCCTACAGGCACTGAGT

AGCACGGCAGCCTCAGGCTCA

HNF4A

GCGGAAGAACCACATGTACTC

GGCTGCTGTCCTCATAGCTT

PPIA

ACCGTGTTCTTCGACATTGC

TTCTGTGAAAGCAGGAACCC

SNAI1

GGTTCTTCTGCGCTACTGCT

TAGGGCTGCTGGAAGGTAAA

SNAI2

GAGCATACAGCCCCATCACT

GGGTCTGAAAGCTTGGACTG

HNF1A

TGCCTCTACTGGGAAGGCTA

CCTGGGGTCACCTCTTTCTT

HNF1B

CACCCCTATGAAGACCCAGA

CCATGGCTCTGTTGACTGAA
